# AS1411-Bivalent-Cholesterol-Anchor Equipped with Zinc Phthalocya-Nine Enables NK Cells Derived Exosomes to Realize Effective Tumor-Tropism Photodynamic Therapy

**DOI:** 10.3390/pharmaceutics18040401

**Published:** 2026-03-24

**Authors:** Yuchen Qi, Haoran Jiang, Yuying Zhang, Zhe Wang, Qianqian Wu, Hua Yu, Boning Xia, Jianjun Li

**Affiliations:** 1 Department of Oncology and Southwest Cancer Center, Southwest Hospital, Third Military Medical University (Army Medical University), Chongqing 400038, China; 2 School of Medical and Life Sciences, Chengdu University of Traditional Chinese Medicine, Chengdu 611137, China; 3 Department of Gastroenterology and Anorectal Surgery, Second Affiliated Hospital of Chongqing Medical University, Chongqing 400010, China

**Keywords:** natural killer cell-derived exosome, tumor-tropism therapy, functional nucleic acids, engineered exosome, photo-dynamic therapy

## Abstract

**Background/Objectives**: Benefiting from their outstanding tumor-penetrating ability and cytotoxic proteins and cytokines, natural-killer-cell-derived exosomes (NEX) show great potential for cell-free tumor immunotherapy. To meet the clinical tumor therapeutic need, engineered NEX are highly required to further enhance their tumor-tropism and antitumor abilities. **Methods**: We proposed a NEX engineering strategy, using a structure of AS1411-bivalent-cholesterol (B-Chol) anchor equipped with photosensitizer zinc phthalocyanine (ZnPc) attached on the membrane of NEX to form A-P-NEX. It not only preferably maintains the spatial structure of the AS1411 aptamer via a B-Chol anchor contributing to the tumor-tropism and stability of NEX but also significantly improves the photodynamic therapy (PDT) effect by firmly binding ZnPc in the unique G-quadruplex structure in the AS1411 aptamer. **Results**: The results showed that A-P-NEX could promote the precise uptake of NEX and ZnPc by tumor cells and produce obvious synergistic NEX-based immunotherapy and PDT upon laser irradiation, demonstrating excellent targeted antitumor effects both in vitro and in vivo. **Conclusions**: This study demonstrates a reliable NEX engineering strategy and paves the way for developing a useful tumor-tropism PDT method.

## 1. Introduction

Hepatocellular carcinoma (HCC) is a global health problem, with morbidity and mortality increasing in recent years. HCC responds poorly to available treatments, with only a third of HCC patients achieving clinical benefit [1,2]. Even in cases of early diagnosis combined with surgical and local treatment measures, HCC still presents poor prognoses. Ultimately, more than half HCC patients will receive systemic therapy [3]. In the past few years, the growing focus on the molecular mechanisms underlying immune response and the interactions among key immune cells in HCC has led to a rising interest in utilizing immunotherapy for its treatment [4].

Natural killer (NK) cells are innate lymphocytes that induce antigen-independent immune responses against malignant cells and function in immune surveillance as the first line of defense against tumor and viral infections [5]. NK cell-derived exosomes (NEX) are nanoscale vesicles (80–120 nm) actively secreted by NK cells. They contain a variety of cytotoxic factors and functional proteins from their parental cells that exhibit good tumor-tropism, killing, and immunomodulatory functions. Many studies in recent years have demonstrated the therapeutic potential of NEX in antitumor therapy [6,7,8]. NEX-based therapy, as an emerging form of cell-free immunotherapy, presents numerous advantages compared to traditional cell therapy. NEX therapeutic potential is attributed to its ability to effectively penetrate and infiltrate tumors at the nanoscale, a characteristic that appears to be maintained even in the face of immunosuppressive tumor microenvironment [9,10,11,12]. In addition, NEX are more economically accessible and present fewer side effects. Thus, NEX-based immunotherapy is expected to overcome the limitations of conventional NK cell-based therapy in the future [13,14].

Although NEX have shown antitumor capabilities, they still have limitations, such as poor stability, erratic targeting, and uptake efficacy. The modification and design of NEX to improve their targeting ability and therapeutic efficacy are now issues of keen interest to many researchers [15,16]. In this study, we aim to enhance the stability and tumor-targeting properties of NEX. Inspired by our previous study, an aptamer AS1411 bivalent-cholesterol-anchor (B-Chol) strategy was proposed for NEX modification [17]. The AS1411 aptamer is a classic 26-nucleotide DNA oligonucleotide that has high affinity and specificity to nucleolin (NCL) [18,19]. NCL is overexpressed in a variety of cancer cells and is an important biomarker for tumor growth and metastasis in HCC [20,21]. Accordingly, it could be used to not only target HCC cells but also inhibit the proliferation of cancer cells [22,23]. AS1411 also shows many advantages compared with traditional antibodies, including its small size, ease of synthesis, flexible chemical modification, low toxicity, and nonimmunogenicity, providing a potential strategy to functionalize exosomes [24]. Our studies demonstrate that the B-Chol structure exhibits superior AS1411-NEX membrane binding efficiency and stability compared to monovalent cholesterol (M-Chol) anchors [17]. M-Chol systems show compromised membrane insertion due to limited single-point hydrophobic interactions, resulting in premature in vivo dissociation. The B-Chol architecture utilizes dual cholesterol units to enhance membrane integration stability through cooperative hydrophobic interactions and van der Waals forces [25].

Photodynamic therapy (PDT) is a clinically adopted noninvasive treatment in which reactive oxygen species (ROS) are generated via the use of photosensitizers and laser irradiation for location-selective damage to target tissues and cells. It is clinically approved by the FDA as one of the effective options for the treatment of tumors and other diseases [26,27]. Zinc phthalocyanine (ZnPc) has a large planar π structure and is used as a photosensitizer in PDT applications. It can be loaded within the unique G-quadruplex structure of AS1411 via π–π stacking to enhance its therapeutic effect.

**Scheme 1:**
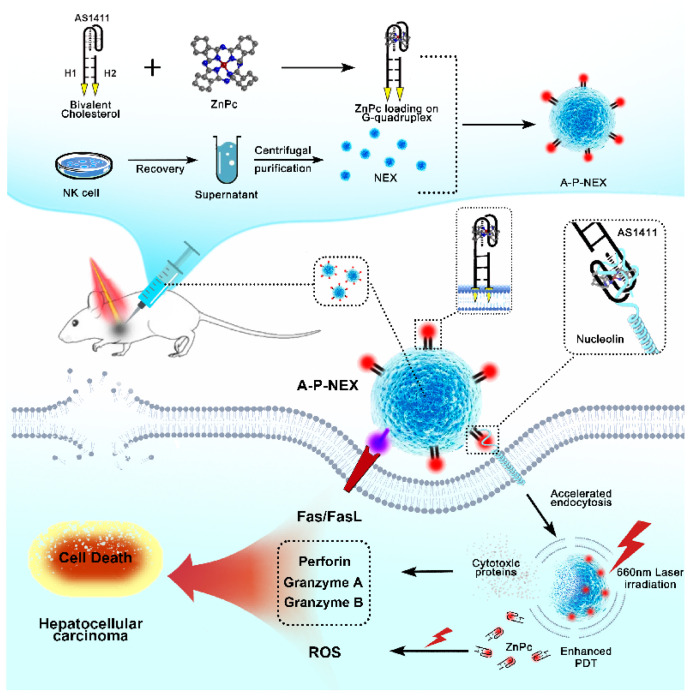
A-P-NEX are formed by anchoring ZnPc-loaded AS1411 to NEX via a B-Chol structure. This modification enhances the targeting of HCC by the NEX and provides a means for accelerated endocytosis and the release of cytotoxic substances. ZnPc induces regulated cell death (apoptosis as the main form) by producing large amounts of ROS under laser irradiation.

In this study, we used a B-Chol anchor structure to modify NEX with ZnPc-loaded AS1411, forming A-P-NEX (Scheme 1). The combination of NEX and photosensitizers offers a promising strategy for tumor-related synergistic PDT-enhanced immunotherapy. This integrative approach, which combines PDT with immunotherapy, represents a mutually beneficial therapeutic strategy for tumor management [28,29].

## 2. Materials and Methods

Cell lines

The human hepatoma cell lines HepG2, Hep3B, Huh7, human NK cell line NK92-MI, and human embryonic kidney 293 (HEK293) were obtained from the American Type Culture Collection (ATCC, Manassas, VA, USA). HepG2, Hep3B, Huh7, and HEK293 cells were cultured in Dulbecco’s Modified Eagle Medium (DMEM; Gibco, Newcastle, NSW, Australia) supplemented with 10% fetal bovine serum (Gibco, Newcastle, NSW, Australia) and 1% penicillin-streptomycin. NK92-MI cells were incubated in stem cell growth medium (iCell Bioscience, Shanghai, China) supplemented with 2% exosome-depleted human serum and 1% penicillin-streptomycin. All cells were cultured at 37 °C in a humidified 5% *v*/*v* CO_2_ atmosphere.

Exosome isolation

NK92-MI-derived exosomes were isolated using differential ultracentrifugation (a classic physical method for separating and purifying exosomes by gradually increasing centrifugal force based on particle size and density). NK92-MI cells were cultured in complete medium for three days, and the supernatant was collected and centrifuged at 300× *g* for 10 min, then at 2000× *g* for 20 min, and then at 10,000× *g* for 30 min at 4 °C. The above steps are followed by discarding the precipitate and retaining the supernatant, followed by ultracentrifugation at 100,000× *g* for 90 min at 4 °C to isolate the exosomes. The precipitated pellets were resuspended in phosphate-buffered saline (PBS), and the protein content was determined using the bicinchoninic acid (BCA) assay.

Western blot analysis

NK cells and NEX samples were prepared for analysis by suspending them in a RIPA lysis buffer. After incubating on ice for a period of 15–30 min (with gentle pipetting every 10 min to ensure thorough lysis), the samples were centrifuged at 20,000× *g* for 20 min. The resulting supernatants were carefully collected and transferred to clean tubes. Quantification of cell and exosome lysates was performed using the BCA assay, and the lysates were then diluted in an LDS sample buffer. Electrophoresis on SDS-page gel 10% (*w*/*v*) was carried out to separate the lysates under either reducing or nonreducing conditions. The separated lysates were subsequently transferred to nitrocellulose filters. To prevent nonspecific binding, the membrane filters were blocked with bovine serum albumin (BSA). Antibodies including: CD63 and TSG101 (exosomal marker proteins), Calnexin (endoplasmic reticulum protein, used as a negative control), Fas-L, perforin, granzyme A, and granzyme B (NK cell-derived cytotoxic effector molecules) (obtained from Bioss, Beijing, China) were then used to incubate the membrane filters at 4 °C overnight with gentle shaking. After primary antibody incubation, the membranes were washed three times with TBST (5 min each). Following this, the membranes were treated with a secondary antirabbit antibody conjugated to horse-radish peroxidase (HRP) for a duration of 1 h at room temperature. The ECL method (Thermo Fisher Scientific, Waltham, MA, USA) was employed to detect the signals, and the optical density was analyzed by using UVI-TEC Nine Alliance analysis software (version 2024).

Transmission Electron Microscope (TEM)

TEM was employed to assess the morphology of the isolated nanoparticles of interest NEX. The NEX samples were immobilized by treating them with a 2% solution of paraformaldehyde. Subsequently, the immobilized NEX were carefully placed onto a transmission electron microscope (JEM-1400; JEOL, Tokyo, Japan) grid that was coated with Formvar and a carbon material (Agar Scientific, Stansted, Essex, UK). Following a 20 min incubation period, the TEM grid was subjected to a brief rinse with a small volume of PBS for a duration of 10 s. Subsequently, the grids were air-dried for a period of 10 min prior to observation under a TEM operating at an acceleration voltage of 80 kV. The resulting images were digitally captured using a Mega View Camera (EMSIS, Münster, Germany).

Nanoparticle Tracking Analysis (NTA)

The size and quantity of the isolated NEX were measured using Nanoparticle Tracking Analysis (NTA) with a Nanosight LM10 system (Malvern Instruments, Malvern, UK) that was equipped with a 405 nm laser. The NEX samples (10 μL) were diluted 100 times with PBS prior to analysis. The NTA data were acquired and processed using Nanosight version 3.1 software provided by Malvern.

Dynamic Light Scattering (DLS)

The exosome size analysis by DLS was conducted using a Zetasizer Nano ZS instrument (Malvern Instruments, Malvern, UK) with a 633 nm laser. Prior to analysis, the samples were placed in a square cuvette with a volume of 1 mL, prepared at a dilution ratio of 1:100 with PBS.

Synthesis and characterization of aptamer AS1411

We designed the structure of the bivalent cholesterol anchor based on the AS1411 aptamer (5′-GGTGGTGGTGGTGGTTGTGGTGGTGGTGGTGG-3′). The structure comprised an A1 (5′-GGTGGTGGTGGTGGTTGTGGTGGTGGTGGTGGTTTTTTGCAGAAATAAGGCACGACGGCTTT-3′) chain and an A2 (5′-CGTCTTTATTCCGTGCTGCCGTTT-3′) chain, which were mixed and slowly annealed to form. In addition, we designed the AS1411 complementary sequence (cDNA: 5′-CCACCACCACCACCAACACCACCACCACCACCTTTTTTGCAGAAATAAGGCACGACGGCTTT-3′) for subsequent experiments. Each chain was modified with a cholesterol structure at the 3′ end. The 5′ end of the A1 chain was modified with FAM fluorescence for subsequent experimental characterization. The oligonucleotides were synthesized by Sangon Biological (Shanghai, China).

Loading of ZnPc on AS1411

In this study, we aimed to investigate the loading of Zinc phthalocyanine (ZnPc) onto aptamer AS1411. To determine the extent of ZnPc loading, we monitored peak increase at 680 nm (the characteristic absorption wavelength of ZnPc, whose absorbance increase correlates with the loading amount of ZnPc on AS1411) using various concentrations of AS1411 (0, 10, 20, 50, and 100 μM). In brief, we mixed 10 μM ZnPc with AS1411 in tris-acetate buffer comprising 0.1 M Na^+^, 25 mM K^+^, and 10 mM Mg^2+^ at pH 7.4. We then measured the peak generation and increase at 680 nm (the characteristic absorption wavelength of ZnPc) using a UV-2550 UV-Vis Spectrophotometer (Shimadzu, Kyoto, Japan); the emergence of the absorption peak indicates the specific binding of ZnPc to the G-quadruplex structure of AS1411, and the increase in absorbance value represents a concentration-dependent binding effect between them. To generate a Job plot (method of continuous variations, a classic spectroscopic method for determining the molar binding stoichiometry between molecules), we prepared mixtures of ZnPc and AS1411 sequence at different molar fractions (0, 0.1, 0.2, 0.4, 0.6, and 0.8 ratio of ZnPc:AS1411). We evaluated the changes in absorbance (Δ absorbance) by determining the ratio of decrease in absorbance peak at 640 nm and increase in absorbance peak at its 680 nm. The amount of loaded ZnPc on AS1411 was determined by processing the Δ absorbance with linear fitting of different molar fractions of ZnPc:AS1411. In addition, we observed the changes in cellular fluorescence in HepG2 cells incubated with ZnPc and AS1411 at various ratios (0, 1:2, 1:3, and 1:4 ratio of ZnPc:AS1411). We inoculated HepG2 cells in 96-well plates and incubated with AS1411-ZnPc for one hour. We then detected cellular fluorescence changes using a Multi-mode Detection Platform (Molecular Devices, Sunnyvale, CA, USA).

Modification of NEX membranes with AS1411-B-Chol

The NEX were incubated with AS1411-B-Chol-FAM (labeled with 5-Carboxyfluorescein (FAM)) and then isolated by differential ultracentrifugation (a classic exosome isolation method). Modification results were determined by fluorescence images taken with a confocal laser scanning microscope (Carl Zeiss, Oberkochen, Germany). Then, equal amounts of NEX (5 μg/mL) were coincubated with different concentrations of AS1411-B-Chol (50–500 nM) for 1 h to explore optimal concentration ratios. The above experiment was accomplished with a Multimode Detection Platform (Molecular Devices, Sunnyvale, CA, USA) and a fluorescence spectrophotometer (F-7000, Hitachi, Tokyo, Japan).

Preparation of A-P-NEX

The A1 chain was mixed 1:1 with the A2 chain in buffer (0.1 M Na^+^, 25 mM K^+^, 10 mM Mg^2+^ at pH 7.4) and annealed by heating to 95 °C for 1 min and then slowly lowering the temperature to 23 °C. ZnPc (5 mg ZnPc dissolved in 2.5 mL DMF under sonication at 100 W for 30 min and stirring at 60 °C overnight, protected from light throughout) was added 1:3 to the synthesized AS1411 and incubated at 37 °C for 1 h, forms a compound of AS1411 and ZnPc. Finally, NEX were added to previously synthesized compound of AS1411 and ZnPc and incubated at 37 °C for 30 min to obtain A-P-NEX.

Cell uptake

To determine the cellular uptake of NEX, AS1411, ZnPc, and A-P-NEX by Hep3B, HepG2, Huh7, and HEK293 cells, the NEX and A-P-NEX were labeled with DiD (red), and AS1411 with FAM (green). Hep3B, HepG2, Huh7, and HEK293 cells were seeded on a coverslip and coincubated with NEX (600 μg/mL), AS1411 (1.8 μM), ZnPc (600 nM), or A-P-NEX (600 μg/mL) at 37 °C for 12 h. Following the incubation period, the cells underwent three consecutive washes with PBS solution and were subsequently fixed for 10 min in a 4% paraformaldehyde solution. The cells were then subjected to an additional three washes with PBS solution and subsequently stained with DAPI, then observed using fluorescence microscopy (Carl Zeiss, Oberkochen, Germany).

As for flow cytometry analysis, cells were digested with trypsin, washed with PBS three times, and suspended in 400 µL PBS. Finally, the fluorescence signals of DiD, FAM, or ZnPc were detected by a FACS Calibur flow cytometer (BD, Franklin Lakes, NJ, USA). Mean fluorescence intensity was quantitatively analyzed by FlowJo software (v10.10).

ROS generation Assay

For the ROS assays, A-P-NEX and ZnPc were co-incubated with HepG2 cells for 12 h. After incubation, cells were stained with DCFH-DA (Beyotime, Shanghai, China) and then treated with laser irradiation at different intensities (0, 10, 20, or 30 J/cm^2^). The results were determined using fluorescence microscopy and flow cytometry.

Cytotoxicity Assay

Cell counting kit-8 (CCK-8), a colorimetric assay for detecting cellular metabolic activity by reflecting the activity of mitochondrial succinate dehydrogenase in living cells, was used to evaluate the cytotoxicity of NEX, AS1411, ZnPc, and A-P-NEX in Hep3B, HepG2, Huh7, and HEK293 cells. Cells were inoculated in clear-bottomed 96-well plates at 1 × 10^4^ cells per well and co-incubated with NEX (200, 400, 600, 800, 1000 μg/mL), AS1411 (1.8 μM), ZnPc (600 nM), or A-P-NEX (200, 400, 600 μg/mL) at 37 °C for 6, 12, or 24 h. Cells were then treated with irradiation under different laser intensities (0, 10, 20 J/cm^2^). After incubation and washing, 10 μL of CCK-8 reagent was then added to the test wells. After incubation, absorbance values at 450 nm (OD450) were measured. The cellular metabolic activity of the co-cultured cells, which is positively correlated with the OD450 value reflecting mitochondrial succinate dehydrogenase activity, was calculated relative to that of control cells. An experiment to evaluate the cytotoxicity of A-P-NEX was also conducted using a Calcein/PI cell viability/cytotoxicity assay kit (Beyotime, Shanghai, China).

Quantification of regulated cell death by flow cytometry

The regulated cell death of HCC cells treated with NEX, AS1411, ZnPc, and A-P-NEX was determined with an Annexin V-FITC kit (Beyotime, Shanghai, China) using a previously reported protocol. HepG2 cells were preincubated with NEX (600 μg/mL), AS1411 (1.8 μM), or A-P-NEX (600 μg/mL) at 37 °C for 12 h. Then, the cells were treated with 20 J/cm^2^ laser irradiation (or not). Cells were digested with trypsin, washed with PBS three times, and suspended in 400 µL PBS. Then, the samples were centrifuged and the supernatant was decanted. The cells were resuspended in the binding buffer at a concentration of 1 × 10^6^ cells/mL. The stain was added and gently mixed with the cells, which were then incubated in the dark for 15 min at room temperature. Binding buffer (400 µL) was added to each tube before analysis by flow cytometry. Data were analyzed using Flowjo software, and the proportions of viable cells (Annexin V-FITC^−^/PI^−^), late-stage dying cells (Annexin V-FITC^+^/PI^+^), and early-stage dying cells (Annexin V-FITC^+^/PI^−^) were quantified.

In vivo animal study

Male BALB/c nude mice (6–8 weeks old, 18–22 g) were obtained from the Experimental Animal Center of Army Medical University and housed under specific pathogen-free conditions with a one-week acclimatization period. All animal procedures were approved by the Institutional Animal Care and Use Committee of Army Medical University (AMUWEC20242060) and conducted in accordance with the NIH Guidelines for the Care and Use of Laboratory Animals. HepG2 cells (2 × 10^6^ cells in 100 μL PBS) were subcutaneously inoculated into the left axilla of each mouse. When tumor volumes reached 70–100 mm^3^, mice were randomly allocated using a random number table into four groups (*n* = 5 per group): (1) PBS, (2) NEX, (3) A-P-NEX, and (4) A-P-NEX + laser (660 nm, 20 J/cm^2^, 100 mW/cm^2^, 200 s). Tumor length (L) and width (W) were measured every other day with digital calipers, and volume was calculated as V = 0.5 × L × W^2^. Body weight was recorded simultaneously. To minimize confounding, cage positions were rotated daily and measurement order was randomized. Group allocation was concealed from the personnel performing tumor measurements and data analysis. Mice were euthanized on day 12 after the first treatment, tumors were excised and weighed, and the tumor inhibition rate was calculated as [1 − (tumor volume of treatment group/tumor volume of control group)] × 100%. Group comparisons were conducted by one-way ANOVA followed by Tukey’s post hoc test, or by Kruskal–Wallis test if parametric assumptions were not met. Results are presented as mean ± SD, and effect sizes with 95% confidence intervals are reported where applicable.

HE, TUNEL Assays and Immunostaining

In this study, standard protocols were employed to perform HE and TUNEL assays on paraffin sections. To fix the sample, a slide was first treated with 4% paraformaldehyde in PBS (pH 7.4) for 10 min at room temperature. The slides were then washed with PBS and incubated in a freshly prepared permeabilization solution containing 0.1% Triton X-100 and 0.1% sodium citrate in water for 2 min. Following washing, tumor samples were incubated with a TUNEL reaction mixture for 60 min at 37 °C in a humidified atmosphere in the dark. After gentle rinsing with PBS, the slides were mounted and prepared for confocal luminescence imaging. Briefly, paraffin tissues were deparaffinized in xylol and rehydrated with gradient ethanol, followed by antigen retrieval. Both frozen and paraffin sections were blocked with 5% goat serum and then incubated with primary antibodies overnight at 4 °C, followed by light-protected incubation with secondary antibodies for 1 h and DAPI for 10 min. For cellular immunostaining, cells were fixed in 4% paraformaldehyde for 15 min, permeabilized with 0.2% Triton X-100 in PBS, and blocked with 5% goat serum, followed by incubation with primary antibodies (Ki-67 Antibody, BD Biosciences, Franklin Lakes, NJ, USA) overnight at 4 °C and secondary antibodies as well as DAPI for 1 h. Quantifications were per-formed by using ImageJ software (1.54p).

Statistical analysis

Experiments were conducted a minimum of three times, and the results are presented as the mean ± standard deviation (SD). Comparisons between two groups were analysed by two-tailed unpaired Student’s *t*-test. Comparisons among multiple groups were analysed by one-way analysis of variance (ANOVA) followed by Tukey’s post hoc test. The significance of the data was shown with *p*-values classified as follows: * *p* < 0.05, ** *p* < 0.01, and *** *p* < 0.001.

## 3. Results

Characterization of NEX

**Figure 1:**
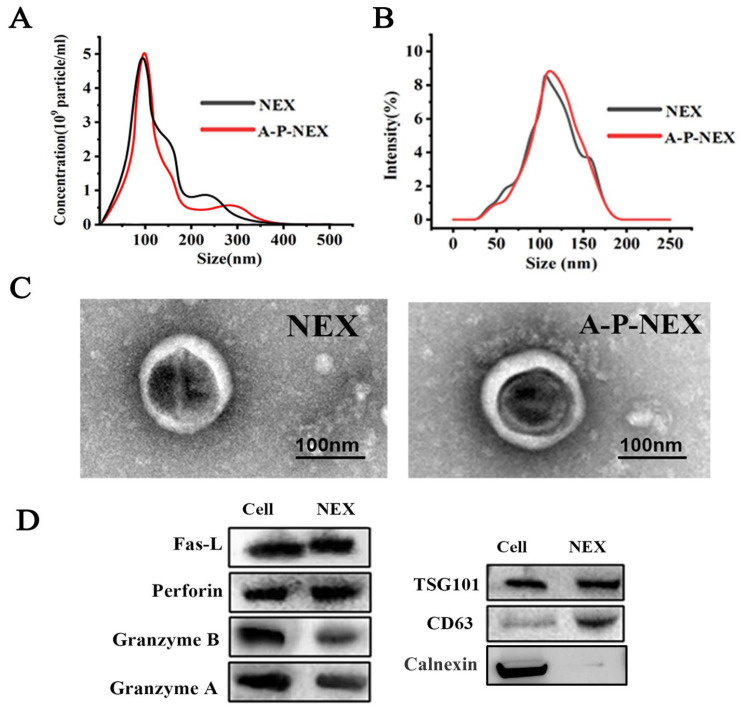
Characterization of NEX. (**A**) Particle-size distributions of NEX and A-P-NEX as revealed by NTA. (**B**) Particle-size distributions of NEX and A-P-NEX as determined by DLS. (**C**) TEM images of a NEX and an A-P-NEX. Scale bar: 100 nm. (**D**) The expressions of NEX markers including CD63, TSG101, and cytotoxic proteins including perforin, granzyme B, granzyme A, and Fas-L were evaluated using Western blotting. Calnexin was used as a negative control. All data are representative of at least three independent experiments.

A wealth of literature has demonstrated that NEX possess potent immunomodulatory properties, which enables them to eradicate tumor cells directly, in addition to eliciting anti-tumor responses through interactions with critical immune effector cells such as T-cells or monocytes [9,30]. To isolate and characterize NEX, supernatants of NK92-MI incubated for three days were subjected to differential ultracentrifugation. The NEX obtained were then subjected to TEM, DLS, NTA, and Western blot analysis. TEM imaging clearly shows that the NEX are homogeneous and spherical with a unique membrane structure and an average size of 110 nm (Figure 1A–C). The specific number and particle-size distributions obtained by NTA show that the NEX are uniformly sized particles with peak diameters of 100–150 nm and a concentration of 5 × 10^9^ particles per mL, which is in accordance with the results of DLS and the defined characteristics of exosomes [31] (Figure 1A,B). In addition, Western blot analysis revealed the presence of the exosomal markers CD63 and TSG101 in NEX, while the negative control protein Calnexin was undetected. These results confirm the successful preparation of high-purity and high-quality NEX. We also determined the toxic proteins (Fas-L, perforin, granzyme A, and granzyme B) in NEX and NK cells by Western blot. The results show that our isolated NEX contain similar toxic proteins to those in the parental cells, which is the biological basis by which NEX exert their tumor-killing effect (Figure 1D).

Assembly of ZnPc and AS1411

**Figure 2:**
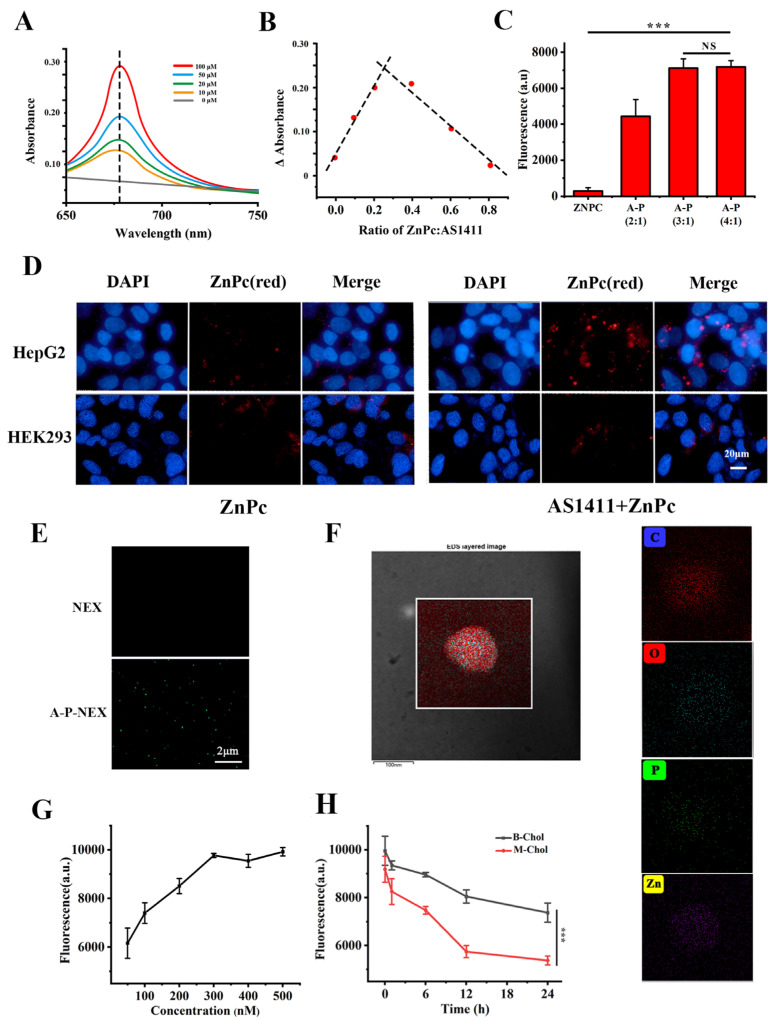
Preparation and performance of A-P-NEX. (**A**) Absorption spectra of different AS1411 concentrations obtained from a mixture of ZnPc and AS1411. (**B**) Job plot for the interaction between ZnPc and AS1411. (**C**) Fluorescence imaging analysis of pris-tine NEX and A-P-NEX after incubation with AS1411-FAM (scale bar: 2 μm). (**D**) Determination of the uptake of ZnPc (red) by HepG2 and HEK293 cells using fluorescence microscopy (ZnPc 600 nM, A-P-NEX 600 μg/mL, scale bar: 20 μm). (**E**) Absorption spectra of different AS1411 concentrations obtained from a mixture of ZnPc and AS1411. (**F**) Job plot for the interaction between ZnPc and AS1411. (**G**) Estimation of the binding ratio of ZnPc to AS1411 by changes in cell fluorescence. (**H**) EDS-mapping images of A-P-NEX. Data are presented as mean ± SD; *n* = 3. *** *p* < 0.001.

The phthalocyanine derivative ZnPc contains a large planar π core that can bind to the G-quadruplex of AS1411 with high specificity and affinity [25]. We introduced ZnPc into the G-quadruplex structure of AS1411 to enable its precise delivery to the target tumor cells. Irradiation of tumor sites using specific wavelengths (660 nm) activates the ZnPc to generate ROS for precise tumor killing. We found that unmodified ZnPc is barely taken up by cells. However, when ZnPc is bound to AS1411, it can be taken up by cells, and the amount taken up can be estimated by detecting the change in cell fluorescence. We confirmed the job plot determined by monitoring the generation of new peaks on the absorbance spectra (Figure 2A). Accordingly, to further test the binding ratio of AS1411 to ZnPc, the cellular uptake efficiency and job plot was conducted (Figure 2B,C), observing results similar to those of previous studies [25,32]. The results of fluorescence microscopy show that ZnPc is internalized by the target cells and that binding to AS1411 improves the internalization efficiency of ZnPc. Furthermore, binding to ZnPc does not affect the original specific targeting ability of AS1411 (Figure 2D).

NEX membrane modification with AS1411-bivalent-cholesterol

In the initial step, the B-Chol structure AS1411 was labelled with fluorescein amidite (FAM) and subsequently co-incubated with NEX. Subsequently, a distinct and pronounced fluorescence emitted by FAM was observed on the surface of NEX, thereby providing conclusive evidence of the successful modification process (Figure 2E). Meanwhile, the successful loading of ZnPc can be confirmed by the EDS-mapping images, in which the Zn signal was originated from ZnPc (Figure 2F). UV visible spectroscopy shows that the absorption of A-P-NEX at long wavelengths (650~750 nm) is significantly stronger than that of NEX, indicating successful loading of ZnPc (Supplementary Figure S1). We then explored several critical issues in the modification of NEX with B-Chol, including the optimal concentration and incubation time for B-Chol to bind to the NEX, as well as the difference in binding efficiency and stability between M-Chol and B-Chol under the same conditions. Our experimental results show that incubation of 5 µg/mL NEX with 300 nM of B-Chol at 37 °C for 30 min as the optimal incubation conditions and used them in all subsequent experiments (Figure 2G, Supplementary Figure S2). Subsequently, we explored the binding efficiencies and stabilities of B-Chol and M-Chol with NEX (Supplementary Figure S3). We then incubated B-Chol-modified and M-Chol-modified NEX in DMEM respectively. Upon measuring the fluorescence change in purified NEX, we concluded that B-Chol provides a higher binding efficiency as well as more stability (Figure 2H). This can be attributed to the robust anchoring force facilitated by the noncovalent interactions between bivalent cholesterol and the lipid membranes of NEX. In addition, we also compared the difference in stability between modified and unmodified NEX by observing changes in NEX morphology by NTA, revealing that the modified NEX exhibit a stronger anti-aggregation ability and can maintain the functional morphology of exosomes more stably (Supplementary Figure S4).

A-P-NEX Enables Precise and Efficient ZnPc Delivery

**Figure 3:**
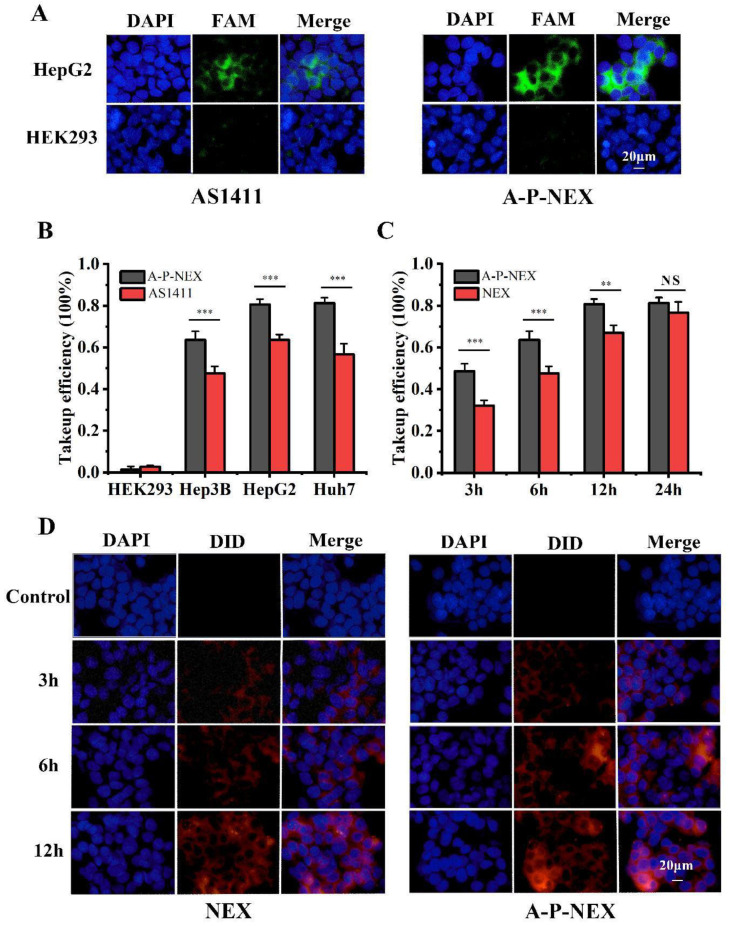
Cellular Uptake of A-P-NEX. (**A**) HepG2 was co-incubated with FAM-labeled AS1411 or A-P-NEX. Fluorescence microscope images were obtained after 12 h coincubation. (**B**) Quantitative flow cytometric analysis of HepG2, Hep3B, Huh7, and HEK293 cells incubated with AS1411 (1.8 μM) or A-P-NEX (600 μg/mL) for 12 h. (**C**) Quantitative flow cytometric analysis of HepG2 incubated with NEX (600 μg/mL) or A-P-NEX (600 μg/mL) for 3, 6, 12, or 24 h; *n* = 3. (**D**) HepG2 was coincubated with NEX or A-P-NEX labeled with the fluorescent dye (DiD). Fluorescence microscopy images were obtained after 3, 6, and 12 h co-incubation. Data are presented as mean ± SD; *n* = 3. ** *p* < 0.01 and *** *p* < 0.001.

The degradation resilience of AS1411 is important to ensure the stable delivery of ZnPc to HCC cells. Accordingly, we labelled AS1411 with FAM, after co-incubation of AS1411 and A-P-NEX (containing the same amount of AS1411) with HepG2, we found by fluorescence microscopy that the fluorescence intensity for the A-P-NEX is higher than that of AS1411 alone (Figure 3C,D). This result suggests that binding to NEX improves the anti-degradation ability and bioavailability of AS1411 and makes it more available to target cells for more efficient delivery of the photosensitizer. Similar results have been reported from previous studies [18,33]. Furthermore, the results of fluorescence microscopy show that ZnPc is internalized by the target cells and that binding to AS1411 improves the internalization efficiency of ZnPc. Furthermore, binding to ZnPc does not affect the original specific targeting ability of AS1411 (Figure 2B). In fact, binding to NEX increases the degradation resilience of AS1411 and maintains its normal morphological structure more stably, ensuring normal loading of ZnPc.

A-P-NEX uptake by HCC

To further validate A-P-NEX preparation and their ability to mediate cellular uptake. we co-incubated cell membrane red fluorescent dye DiD-labeled NEX or DiD-labeled A-P-NEX with HepG2 for 3, 6, or 12 h. The results show that both NEX and A-P-NEX are taken up by HepG2, but A-P-NEX are more efficiently taken up by HepG2, reaching peak uptake at 12 h (Figure 3A,B). This indicates that NEX alone have certain targeting properties but that the membrane-modification strategy we designed enhances their uptake by HCC. Finally, we labeled AS1411 with FAM and NEX with DiD. The experimental group (EG) was NEX pre-combined with AS1411, and the control group (CG) was not combined. EG and CG samples were co-incubated with HepG2 for 6 h, and the resultant cell fluorescence was detected using flow cytometry. The results show that the green (FAM) and red (DiD) signals for the EG are stronger than those of the CG. This indicates that the increased uptake efficiency of A-P-NEX is due to membrane modification (Supplementary Figure S5). We observed that unmodified NEX have the tumor-targeting ability, but membrane modification of NEX with AS1411 enhances their targeting and uptake efficiency and mediates the endocytosis of ZnPc to achieve precise PDT, inhibiting tumor cell proliferation.

Effects of ROS production on tumor cell killing

**Figure 4:**
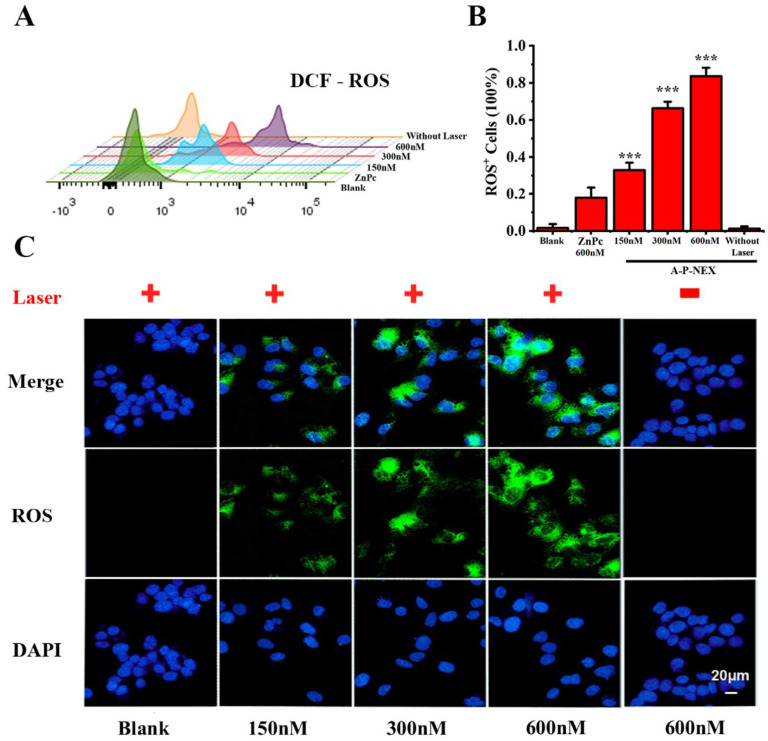
Photogeneration and biological effects of ROS in A-P-NEX-treated HCC. (**A**) ROS production in HepG2 cells after co-incubation with A-P-NEX at different concentrations (and with ZnPc alone) upon laser irradiation. (**B**) Quantitative analysis of data in (**A**) (*n* = 3). (**C**) ROS levels in HepG2 cells after treatment with A-P-NEX (loaded ZnPc concentrations 150, 300, and 600 nM). ROS stained with DCFH-DA. Scale bar: 20 µm. Data are presented as mean ± SD; *n* = 3. *** *p* < 0.001.

We confirmed ROS production using fluorescence microscopy and fluorescence-activated cell sorting (FACS). As shown in Figure 4A,B and Figure S6, FACS shows concentration- and irradiation-dependent production of ROS, and the photosensitizer ZnPc combined with NEX is more efficient in generating ROS under the same conditions. We also observed similar results by fluorescence microscopy, which confirmed concentration- and irradiation-dependent production of ROS (Figure 4C). Next, we examined the impact of ZnPc loading onto AS1411 with regard to ROS production. Specifically, we utilized a singlet oxygen sensor green to monitor singlet oxygen levels generated by photosensitizers. Our findings reveal that the use of AS1411 sequence for ZnPc loading results in a noteworthy increase in singlet oxygen levels compared to the levels produced by free ZnPc. Importantly, we observed that a random DNA sequence does not have the same effect on singlet oxygen production, thus emphasizing the significance of the G-quadruplex sequence in AS1411 for the sequence-specific loading of ZnPc (Supplementary Figure S7).

Cytotoxicity of A-P-NEX against HCC

**Figure 5:**
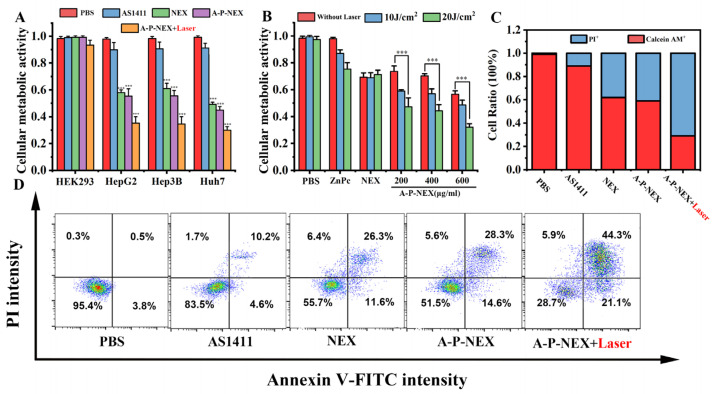
In vitro killing effect of A-P-NEX on HCC. (**A**) Relative cellular metabolic activity (reflected by OD450 value of CCK-8 assay) of HepG2, Hep3B, Huh7, and HEK293 cells after treatment with NEX with different modifications, measured by CCK-8 assay. (**B**) Relative cellular metabolic activity (reflected by OD450 value of CCK-8 assay) of HepG2 cells after treatment with NEX with different modifications at different concentrations and laser intensities, measured by CCK-8 assay. (**C**) FACS results showing cell viability and cytotoxicity in HepG2 cells after treatment using NEX with different modifications (AS1411: 600 nM; NEX: 600 μg/mL; A-P-NEX: 600 μg/mL; A-P-NEX+L: 600 μg/mL with 20 J/cm^2^ laser irradiation. The cells were stained with Calcein AM/PI. (**D**) FACS results showing regulated cell death (early and late stages) in HepG2 cells after treatment with AS1411, NEX, or A-P-NEX. The cells were stained with Annexin V-FITC and PI, with quadrants representing viable cells (Annexin V-FITC^−^/PI^−^), early-stage dying cells (Annexin V-FITC^+^/PI^−^) and late-stage dying cells (Annexin V-FITC^+^/PI^+^). Data are presented as mean ± SD; *n* = 3. *** *p* < 0.001.

To verify the killing effects of NEX and A-P-NEX on HCC cells, we co-incubated Hep3B, HepG2, Huh7, and HEK293 with NEX or A-P-NEX at different concentrations. The results show that NEX achieve their maximal anti-tumor effect at a concentration of 600 µg/mL after 12 h (Supplementary Figures S8 and S9). On this basis, we measured the anti-tumor effects of the A-P-NEX and explored the relationships between these effects and laser intensity and photosensitizer concentration. The concentration of ZnPc used in the experiment was 600 nM, the concentration of NEX was 600 µg/mL, and the concentration of A-P-NEX was 200, 400, or 600 μg/mL. The results showed that variations in both laser intensity and photosensitizer concentration affect the antitumor effect, and that A-P-NEX exhibit superior ability to inhibit the cellular metabolic activity of HCC cells (Figure 5A,B). A clonogenic assay further validated the long-term anti-proliferative effect of A-P-NEX, and the results revealed that A-P-NEX combined with laser irradiation almost completely abolished the colony formation of HepG2 cells while exerting no obvious influence on normal HEK293 cells (Supplementary Figure S10).

Subsequently, we measured the activity of Hep3B, HepG2, Huh7, and HEK293 cells after incubation with A-P-NEX to explore the effects of our modifications on the antitumor activity of NEX. The results showed that the modified NEX decrease the cellular metabolic activity of all three HCC cell lines in a concentration- and time-dependent manner (Figure 5A). Furthermore, the above results were validated using Calcein AM/PI (Figure 5C). Finally, our experimental results showed that the NEX have no effect on the cellular metabolic activity of normal HEK293 cells, regardless of concentration or modification (Figure 5A,C). We chose Annexin V-FITC to stain HepG2 cells treated with A-P-NEX, and after successful staining, the cells were analyzed and quantified by flow cytometry for live cells (Annexin V-FITC^−^/PI^−^), necrotic cells (Annexin V-FITC^+^/PI^+^), and apoptotic cells (Annexin V-FITC^+^/PI^−^). The results showed that both NEX and A-P-NEX induced regulated cell death in HepG2 cells in vitro, and that the proportion of regulated cell death was significantly increased by combination with PDT (Figure 5D). A-P-NEX leverages the tumor-penetrating capabilities and cytotoxic payload (e.g., granzyme B, perforin) of its parental NEX, leading to enhanced cell death through a dual mechanism. Specifically, the cytotoxic proteins within NEX, such as granzyme B and perforin, act synergistically with PDT-induced reactive oxygen species (ROS). This synergy is achieved via two complementary pathways, one of which involves ROS-mediated membrane destabilization. Laser-induced ROS generation disrupts the integrity of lysosomal and mitochondrial membranes, thereby facilitating the entry of granzyme and perforin into the cytosol, culminating in the activation of caspase-dependent cell death (apoptosis)—the predominant form of regulated cell death in this experimental system.

In vivo antitumor effect of A-P-NEX

**Figure 6:**
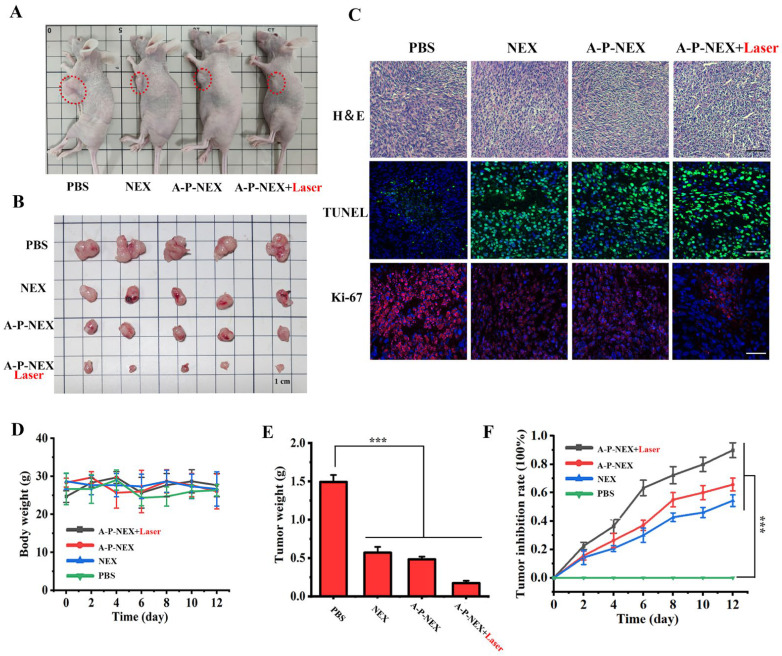
In vivo killing effect of A-P-NEX on HCC. (**A**,**B**) Representative images of tumors in HCC-bearing mice treated with PBS (blank), NEX (800 μg), A-P-NEX (800 μg), A-P-NEX+L (800 μg, laser intensity: 20 J/cm^2^ (power density: 100 mW/cm^2^ irradiation duration: 200 s)). (**C**) Immunofluorescent and histology staining of the tumor tissue. Scale bar: 100 µm. (**D**–**F**) Changes in body weight (**D**), tumor weight (**E**), and tumor-inhibition rate (**F**) for HCC-bearing mice following different treatments. Body weight was measured until 12 days. The tumor inhibition rate was calculated as [1 − (tumor volume of treatment group/tumor volume of control group)] × 100 (%). Data are presented as mean ± SD; *n* = 5. *** *p* < 0.001.

To evaluate the in vivo therapeutic effects of A-P-NEX against HCC, we established a xenograft mouse model. We determined that NEX, A-P-NEX, and A-P-NEX+L (A-P-NEX followed by laser irradiation, 660 nm, 20 J/cm^2^) treatments act as cytotoxic regulators to provide anti-tumor activity (Supplementary Figure S11). The tumor growth is de-scribed by the size of the tumor in each group (Figure 6A,B). The A-P-NEX+L group showed the strongest tumor suppression rate and the smallest tumor weight after treatment (Figure 6E,F). Furthermore, histological examination using hematoxylin and eosin (H&E) staining revealed substantial hypocellularity and necrosis within the tumor xenografts of the A-P-NEX+L cohort. These tumor specimens exhibited elevated TUNEL-positive cell counts and markedly reduced expression of the proliferation biomarker Ki-67. These findings suggest that A-P-NEX+L treatment impede tumor growth and induce apoptotic cell death (Figure 6C). No significant differences in body weights were observed (Figure 6D). Thus, these results demonstrate that A-P-NEX+L treatment significantly inhibits the growth of HCC in vivo, providing strong support for its therapeutic effect.

## 4. Discussion

The proposed A-P-NEX employs a B-Chol anchoring strategy to facilitate the efficient delivery of ZnPc to nucleolin-overexpressing liver cancer cells, while preserving the structural and functional integrity of the NEX membrane. Our comprehensive characterization of native NEX confirmed these vesicles are high-purity spherical exosomes with a typical size distribution of 100–150 nm in peak diameter and intact membrane architecture, which endogenously express cytotoxic proteins (perforin, granzyme A/B, Fas-L) consistent with parental NK92-MI cells. These inherent properties lay a solid biological foundation for the tumor-killing capacity of A-P-NEX, and the noncovalent B-Chol modification further ensures the intrinsic functions of NEX remain unimpaired, in line with our modification optimization results. Upon irradiation with a 660 nm laser, A-P-NEX mediates the locally enhanced generation of ROS to induce tumor cell regulated cell death (apoptosis as the predominant form) and elicit synergistic photo-immunotherapeutic effects; our in vitro assays verified this ROS production exhibits distinct concentration- and irradiation-dependence, and the specific G-quadruplex binding of AS1411 to ZnPc significantly elevates singlet oxygen levels compared to free ZnPc, a key finding that underpins efficient photodynamic tumor killing.

In contrast to conventional PDT approaches, A-P-NEX exhibit markedly reduced off-target toxicity and augmented anti-tumor efficacy. In vitro experiments demonstrated that A-P-NEX exerts concentration- and time-dependent inhibitory effects on the cellular metabolic activity of multiple HCC cell lines (Hep3B, HepG2, Huh7) with superior activity over unmodified NEX, while showing no cytotoxicity to normal HEK293 cells at all tested concentrations. In vivo xenograft mouse model results further validated that the A-P-NEX + 660 nm laser group achieved the strongest tumor suppression rate and the smallest tumor weight; H&E staining revealed extensive tumor necrosis in this group, accompanied by elevated TUNEL-positive apoptotic cells and reduced expression of the proliferation marker Ki-67 in tumor tissues. A clonogenic assay further complemented these findings by verifying the long-term anti-proliferative effect of A-P-NEX + laser irradiation on HepG2 cells with complete abrogation of colony formation, while maintaining negligible influence on normal HEK293 cells. Notably, no significant changes in mouse body weight were observed during the experiment, fully confirming the platform’s high tumor specificity and favorable in vivo biocompatibility. The enhanced anti-tumor effect of A-P-NEX is attributed to its unique dual killing mechanism verified in our experiments: the endogenous cytotoxic proteins of NEX act synergistically with PDT-induced ROS, where laser-generated ROS disrupts lysosomal and mitochondrial membrane integrity, promoting the cytosolic entry of granzyme B and perforin and subsequent activation of caspase-dependent cell death (apoptosis)—the major form of regulated cell death observed in our study.

However, the clinical translation of A-P-NEX requires further investigation of several practical aspects closely linked to our established preparation and modification processes. First, it is imperative to develop scalable methods for large-scale production of clinical-grade NK-exosomes—our current differential ultracentrifugation method is suitable for laboratory-scale preparation but lacks the efficiency required for clinical application, necessitating the optimization of industrialized preparation processes. Second, the long-term storage stability of A-P-NEX under clinically relevant conditions needs to be further optimized; although our experiments confirmed B-Chol-modified NEX have stronger anti-aggregation ability than unmodified and M-Chol-modified NEX, the stability of A-P-NEX under low-temperature, freeze–thaw and other clinical storage conditions remains uncharacterized. Third, a comprehensive assessment of the immunogenicity of the engineered A-P-NEX complex is warranted: despite the low immunogenicity of the AS1411 aptamer itself, A-P-NEX is a multi-component complex composed of NEX, AS1411-B-Chol and ZnPc, and its potential immune responses in the human body need in-depth evaluation.

The clinical translatability of this platform is strongly underpinned by its amenability to scalable engineering methodologies, avoidance of complex chemical modifications, and promising potential for synergistic treatment with immune checkpoint inhibitors. Our established B-Chol anchoring strategy is a simple and efficient noncovalent modification method, with the optimal conditions (5 µg/mL NEX incubated with 300 nM B-Chol at 37 °C for 30 min) enabling stable membrane modification without complex chemical cross-linking—this facile process is highly conducive to subsequent scale-up production. Moreover, NEX inherently possess potent immunomodulatory properties that can elicit anti-tumor immune responses by interacting with key immune effector cells such as T cells and monocytes, making A-P-NEX an ideal candidate for combination with immune checkpoint inhibitors to reverse tumor immune escape and amplify systemic anti-tumor effects. Future investigations will focus on optimizing the long-term storage stability of A-P-NEX and validating its adaptability across diverse nucleolin-overexpressing cancer types, thereby establishing a feasible paradigm for precision tumor therapy. Specifically, subsequent studies will optimize the preservation conditions of A-P-NEX to maintain its structural and functional integrity for clinical use, and verify its therapeutic efficacy in other solid tumors with nucleolin overexpression, aiming to develop a universal precision therapy platform integrating targeted delivery, PDT and immunotherapy.

## Data Availability

The data that support the findings of this study are available in the Supplementary Materials of this article.

## Supplementary Materials

The following supporting information can be downloaded at https://www.mdpi.com/article/10.3390/pharmaceutics18040401/s1. Figure S1: The absorption and emission spectra of NEX and A-P-NEX. Figure S2: The optimal incubation time for NEX binding to B-Chol, as determined by changes in fluorescence. Figure S3: Verification of the binding efficiency of B-Chol and M-Chol to NEX by measuring the fluorescence of purified NEX. Data are presented as mean ± SD; *n* = 3. *** *p* < 0.001. Figure S4: NEX stability as determined by measuring the change in NEX particle size dispersion over 48 h (DLS measures the size of NEX particles and quantifies the degree of dispersion by calculating variance). Data are presented as mean ± SD; *n* = 3. ** *p* < 0.01 and *** *p* < 0.001. Figure S5: Quantitative flow cytometric analysis of HepG2 cells incubated under ex-perimental (EG) and control group (CG) conditions. The experimental group (EG) was NEX pre-combined with AS1411, and the control group (CG) was not combined. Data are presented as mean ± SD; *n* = 3. *** *p* < 0.001. Figure S6: Effect of different laser intensities on ROS production. Data are presented as mean ± SD; *n* = 3. *** *p* < 0.001. Singlet Oxygen Generation test. The present study aimed to investigate the singlet oxygen generation from ZnPc-loaded DNA. Three samples were prepared for this purpose, including free ZnPc, ZnPc-loaded AS1411 sequence, and ZnPc-loaded AS1411 complementary (cDNA) sequence. The samples were made in a phosphate buffer solution containing 0.1 M Na^+^, 25 mM K^+^, and 10 mM Mg^2+^ at pH 7.4. The concentration of ZnPc was 600 nM, and the DNA concentration was 1.8 μM. The singlet oxygen sensor green (SOSG) was added to each sample with a final concentration of 200 nM. A 660 nm laser was used at a power density of 20 J/cm^2^ for laser irradiation. The fluorescence of the samples was measured at an excitation wavelength of 504 nm and an emission wavelength of 525 nm after laser irradiation. Figure S7: Singlet oxygen generation from free ZnPc, ZnPc-loaded AS1411 sequence, and ZnPc-loaded AS1411 complementary (cDNA) sequence. Data are presented as mean ± SD; *n* = 3. *** *p* < 0.001. Figure S8: Relative cellular metabolic activity (reflected by OD450 value of CCK-8 as-say) of HepG2, Hep3B, Huh7 and HEK293 cells after 12 h co-incubation with different concentrations of NEX (200, 400, 600, 800, 1000 µg/mL), measured by CCK-8 assay. Data are presented as mean ± SD; *n* = 3. *** *p* < 0.001. Figure S9: Relative cellular metabolic activity (reflected by OD450 value of CCK-8 as-say) of HepG2 cells after 24 h incubation with AS1411 (1.8 μM) or unmodi-fied/modified NEX (600 μg/mL) with or without 20 J/cm^2^ laser irradiation, measured by CCK-8 assay. Data are presented as mean ± SD; *n* = 3. *** *p* < 0.001. Clonogenic Assay. HepG2 and HEK293 cells were seeded in 6-well plates at 1000 cells per well and incubated overnight at 37 °C with 5% CO_2_ for adherence. Cells were then treated with PBS, AS1411 (1.8 μM), NEX (600 μg/mL), A-P-NEX (600 μg/mL), or A-P-NEX (600 μg/mL) combined with 20 J/cm^2^ laser irradiation, consistent with the in vitro PDT conditions. After continuous culture for 10–14 days, colonies were fixed with 4% paraformaldehyde, stained with 0.1% crystal violet, and non-specific staining was washed off with deionized water. Stained colonies were photographed, and visible colonies (≥50 cells) were counted. All experiments were performed in triplicate with three independent repetitions. Figure S10: Clonogenic assay of HepG2 and HEK293 cells after different treatments. Representative crystal violet-stained images of cell colonies formed by HepG2 and HEK293 cells treated with PBS, AS1411 (1.8 μM), NEX (600 μg/mL), A-P-NEX (600 μg/mL) or A-P-NEX (600 μg/mL) plus 20 J/cm^2^ laser irradiation (*n* = 3). Figure S11: Schematic diagram of treatment plan: Inoculate Hep G2 cells into the ax-illa of mice and inject different modified NEX. PDT is achieved by irradiating with a 660 nm laser (power density: 100 mW/cm^2^, irradiation duration: 200 s).

## Abbreviations

The following abbreviations are used in this manuscript:

NEX	Natural-killer-cell-derived exosomes
A-P-NEX	AS1411-ZNPC-natural-killer-cell-derived exosomes
B-Chol	AS1411-bivalent-cholesterol
ZnPc	Zinc phthalocyanine
AS1411	Antisense oligonucleotides
PDT	Photodynamic therapy
HCC	Hepatocellular carcinoma
NK	Natural killer
NCL	Nucleolin
DNA	Deoxyribonucleic acid
M-Chol	Monovalent cholesterol
ROS	Reactive oxygen species
HepG2	Human hepatocellular carcinoma cell line G2
Hep3B	Human hepatocellular carcinoma cell line 3B
Huh7	Human hepatoma cell line 7
NK92-MI	Natural killer 92, membrane interleukin-2 receptor modified
HEK293	Human embryonic kidney 293
ATCC	American type culture collection
CO_2_	Carbon dioxide
DMEM	Dulbecco’s modified eagle medium
PBS	Phosphate-buffered saline
BCA	Bicinchoninic acid
CD63	Cluster of differentiation 63
TSG101	Tumor susceptibility gene 101
Calnexin	Calcium-binding protein calnexin
Fas-L	Fas ligand
ECL	Enhanced chemiluminescence
HRP	Horseradish peroxidase
TEM	Transmission electron microscope
NTA	Nanoparticle tracking analysis
DLS	Dynamic light scattering
FAM	5-Carboxyfluorescein
DiD	1,1′-Dioctadecyl-3,3,3′,3′-tetramethylindodicarbocyanine perchlorate
CCK-8	Cell counting kit-8
OD	Optical density
SD	Standard deviation
ANOVA	One-way analysis of variance
EG	Experimental group
CG	Control group
FACS	Fluorescence-activated cell sorting
H&E	Hematoxylin and eosin
