# Supplementary figures and images for: AS1411-Bivalent-Cholesterol-Anchor Equipped with Zinc Phthalocya-Nine Enables NK Cells Derived Exosomes to Realize Effective Tumor-Tropism Photodynamic Therapy
Source: Pharmaceutics. 2026 Mar 24;18(4):401. doi: 10.3390/pharmaceutics18040401 (PMC13118968; doi:10.3390/pharmaceutics18040401)

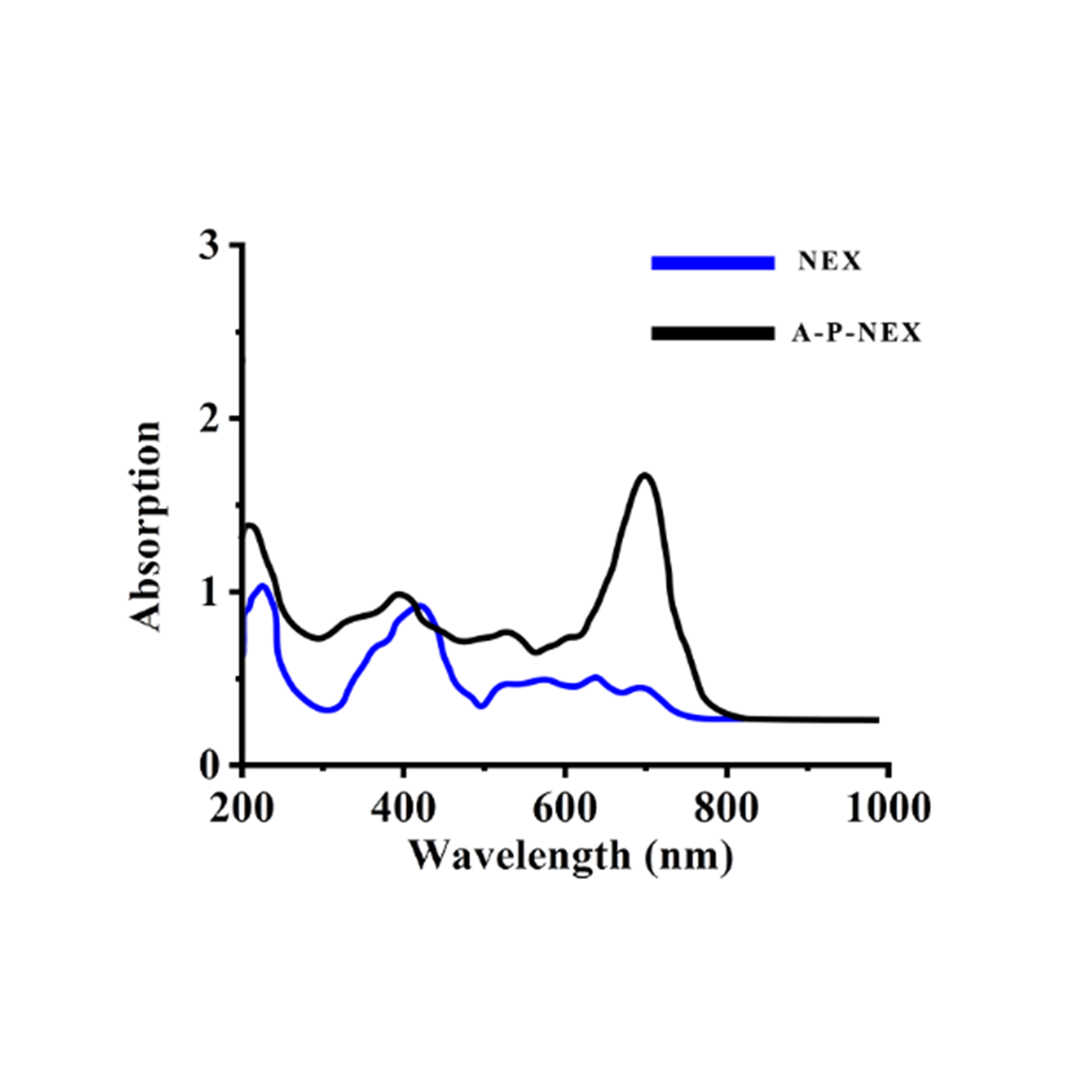

Supplement: Supplementary file 1 [file pharmaceutics-18-00401-s001.zip › Figure S1.PNG]

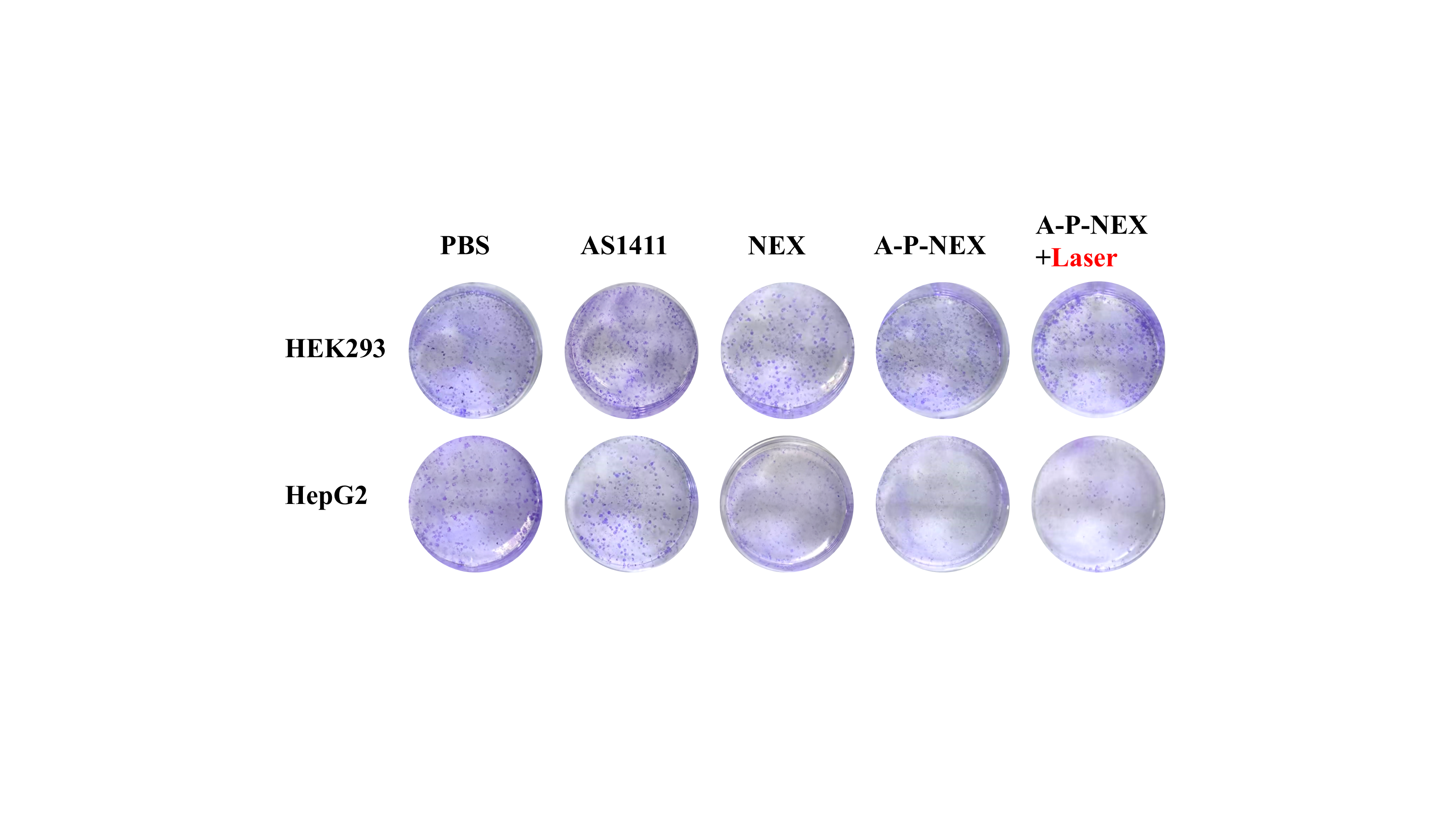

Supplement: Supplementary file 1 [file pharmaceutics-18-00401-s001.zip › Figure S10.PNG]

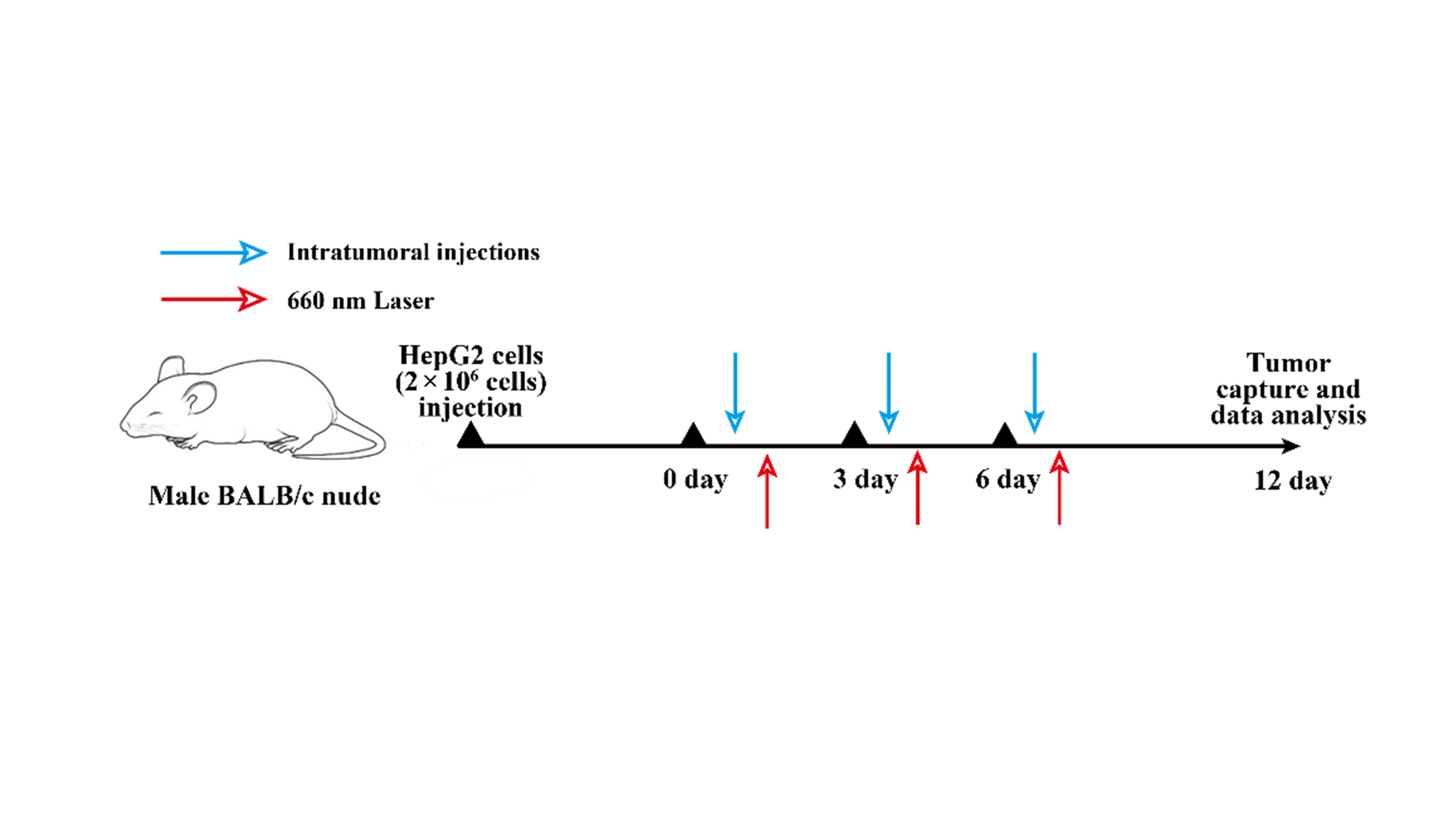

Supplement: Supplementary file 1 [file pharmaceutics-18-00401-s001.zip › Figure S11.PNG]

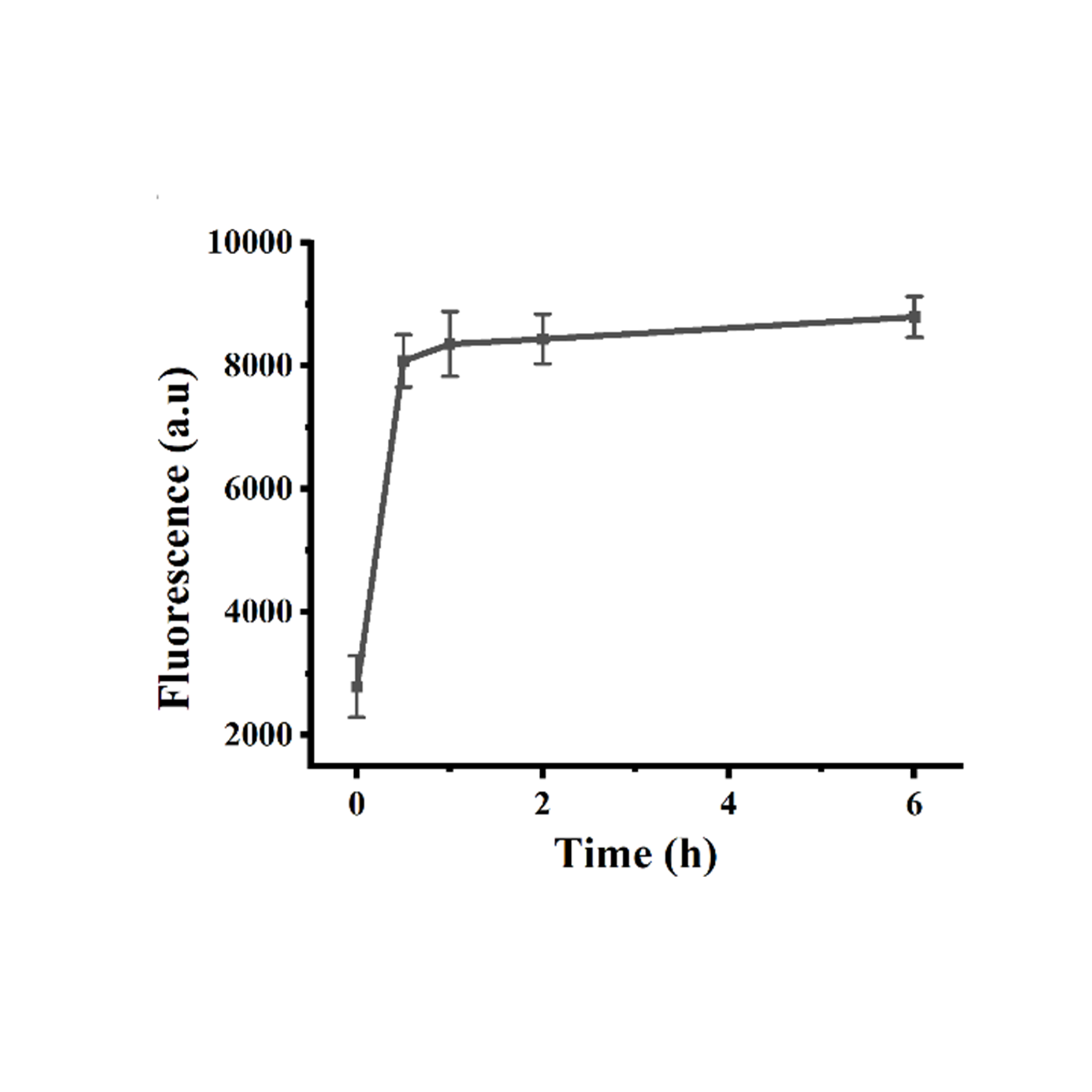

Supplement: Supplementary file 1 [file pharmaceutics-18-00401-s001.zip › Figure S2.PNG]

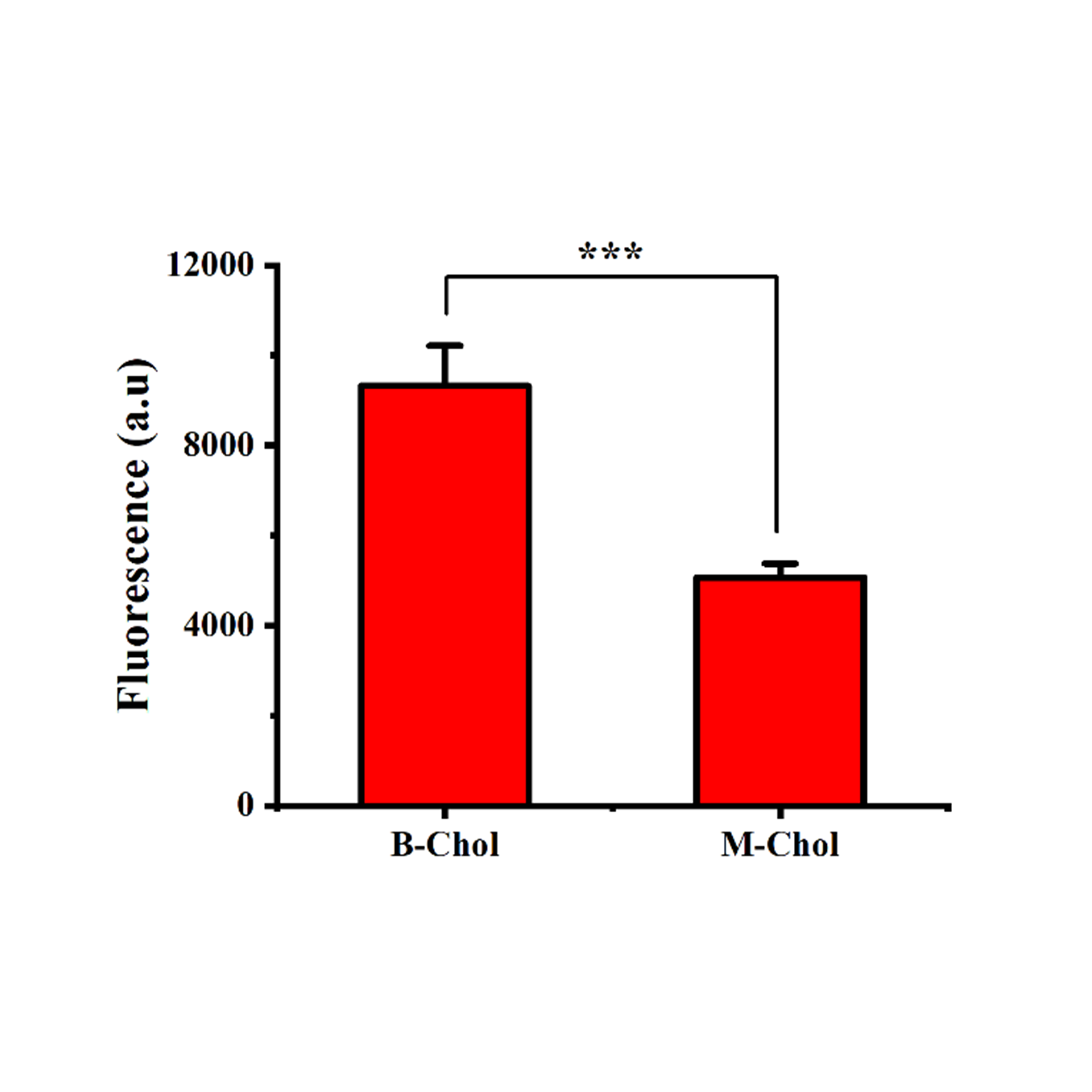

Supplement: Supplementary file 1 [file pharmaceutics-18-00401-s001.zip › Figure S3.PNG]

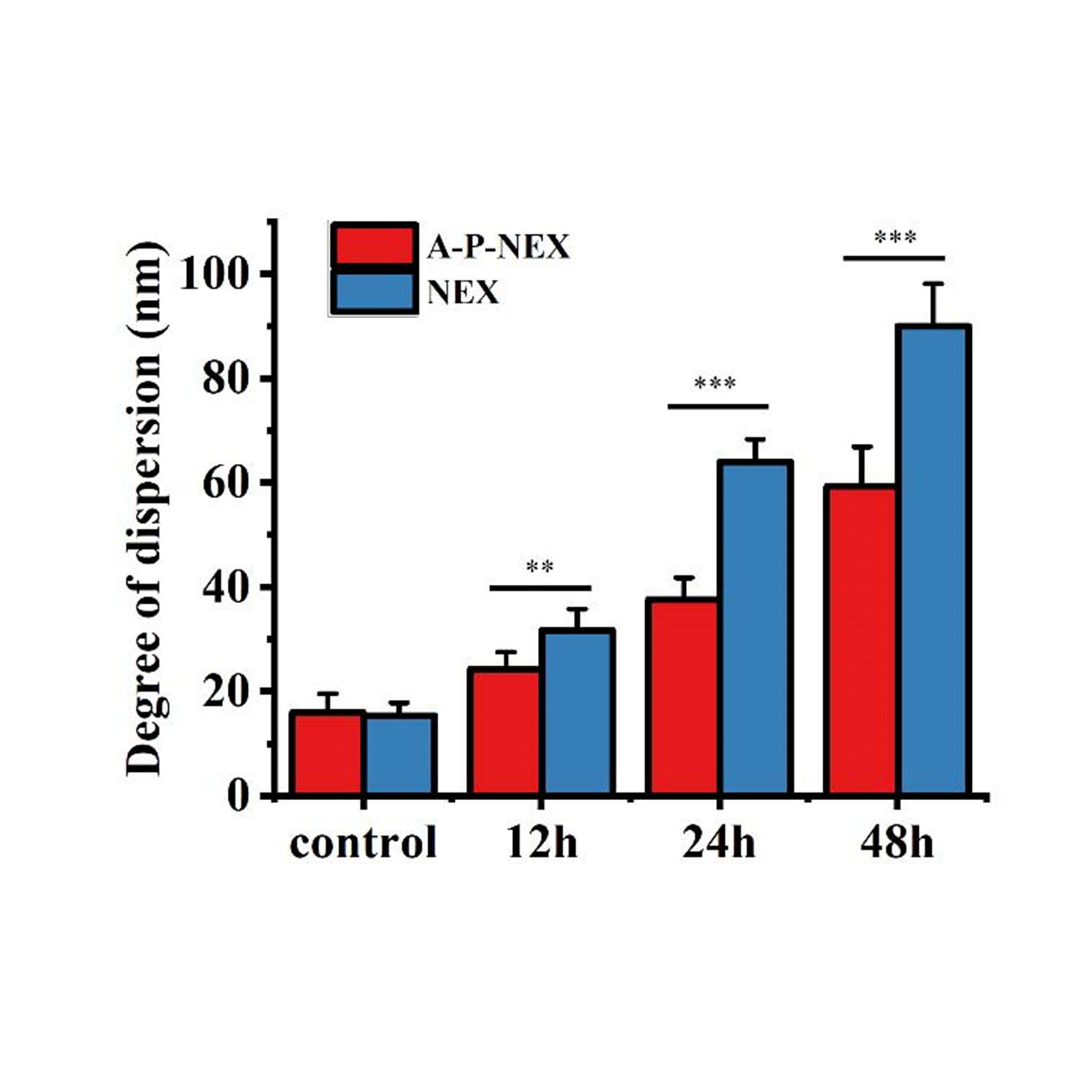

Supplement: Supplementary file 1 [file pharmaceutics-18-00401-s001.zip › Figure S4.PNG]

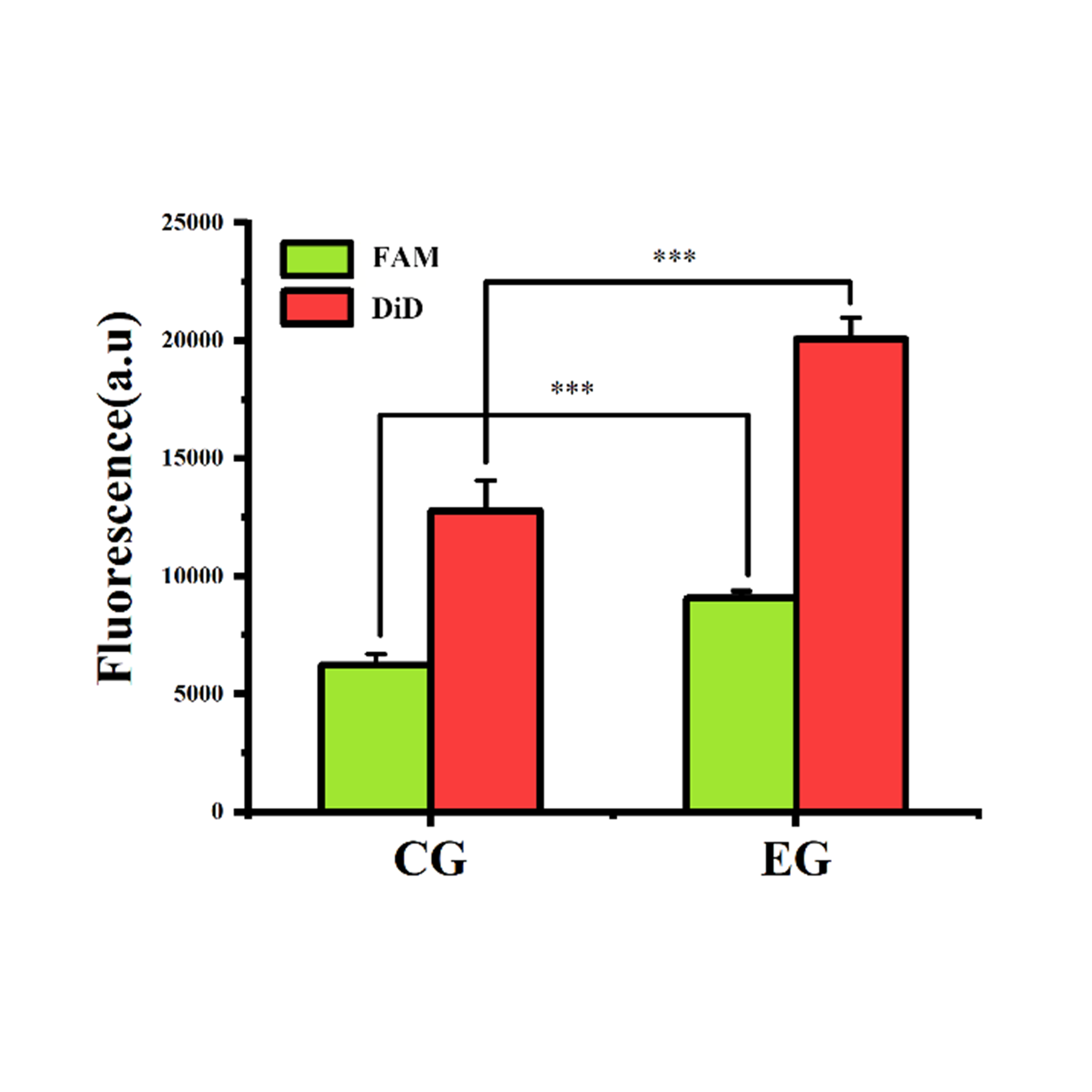

Supplement: Supplementary file 1 [file pharmaceutics-18-00401-s001.zip › Figure S5.PNG]

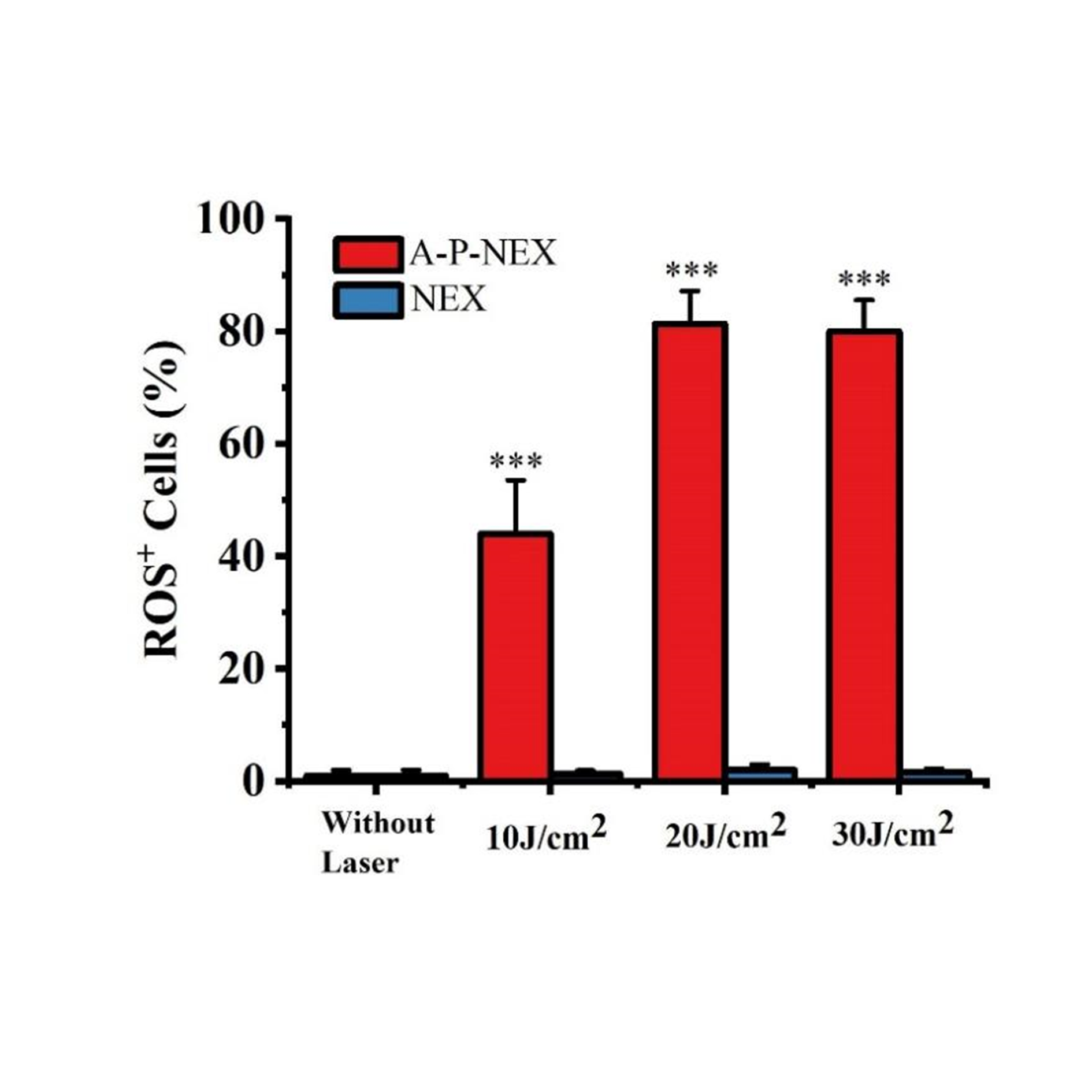

Supplement: Supplementary file 1 [file pharmaceutics-18-00401-s001.zip › Figure S6.PNG]

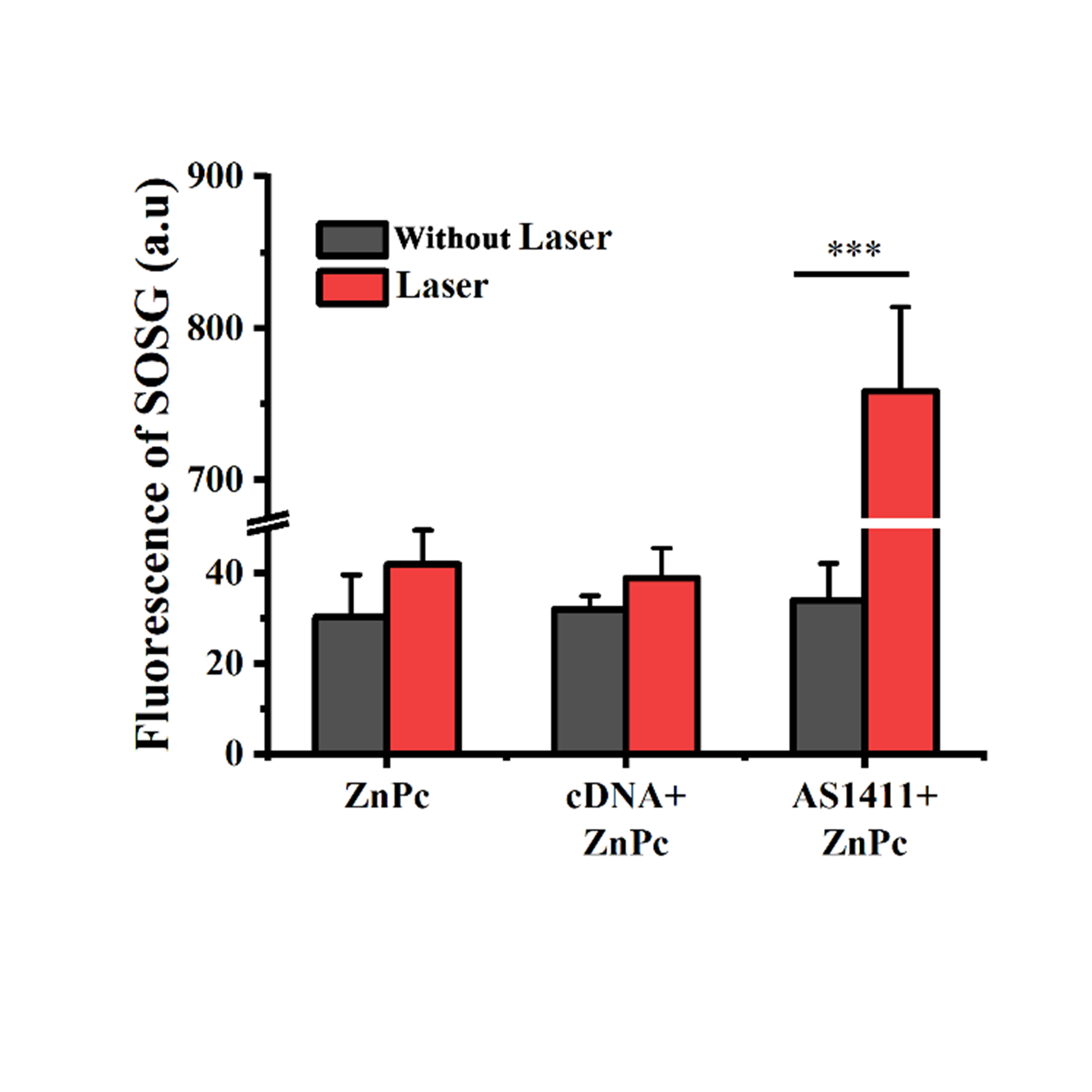

Supplement: Supplementary file 1 [file pharmaceutics-18-00401-s001.zip › Figure S7.PNG]

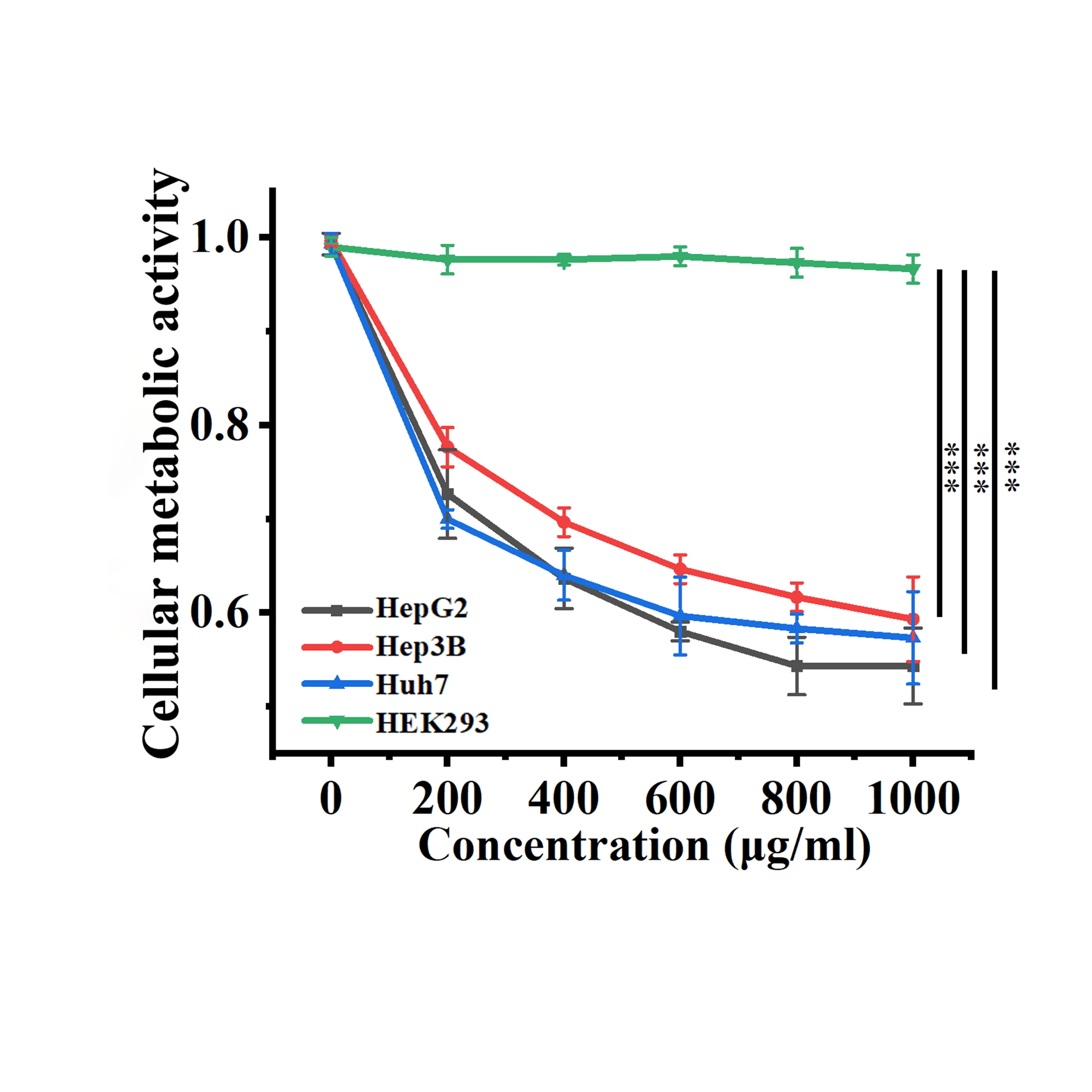

Supplement: Supplementary file 1 [file pharmaceutics-18-00401-s001.zip › Figure S8.PNG]

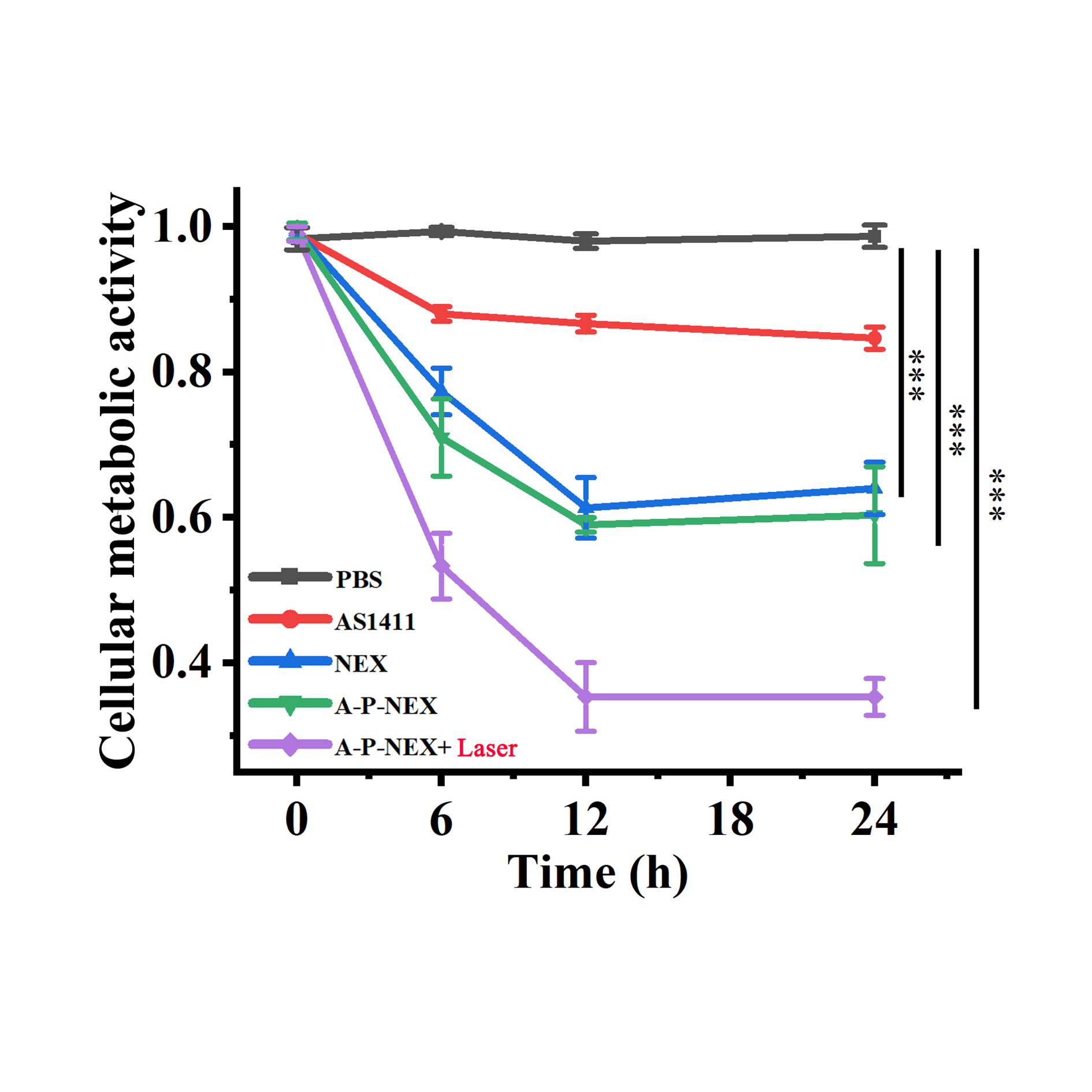

Supplement: Supplementary file 1 [file pharmaceutics-18-00401-s001.zip › Figure S9.PNG]
